# Metabolomic profile of diet-induced obesity mice in response to humanin and small humanin-like peptide 2 treatment

**DOI:** 10.1007/s11306-019-1549-7

**Published:** 2019-06-06

**Authors:** Hemal H. Mehta, Jialin Xiao, Ricardo Ramirez, Brendan Miller, Su-Jeong Kim, Pinchas Cohen, Kelvin Yen

**Affiliations:** Leonard Davis School of Gerontology, University of Southern California, Los Angeles, CA USA

**Keywords:** Mitochondrial-derived peptide, Obesity, Insulin resistance, Aging, UPLC-MS/MS, Sphingolipids

## Abstract

**Introduction:**

The mitochondrial-derived peptides (MDPs) are a novel group of natural occurring peptides that have important signaling functions and biological activity. Both humanin and small-humanin-like peptide 2 (SHLP2) have been reported to act as insulin sensitizers and modulate metabolism.

**Objectives:**

By using a metabolomic approach, this study explores how the plasma metabolite profile is regulated in response to humanin and SHLP2 treatment in a diet-induced obesity (DIO) mouse model. The results also shed light on the potential mechanism underlying MDPs’ insulin sensitization effects.

**Methods:**

Plasma samples were obtained from DIO mice subjected to vehicle (water) treatment, or peptide treatment with either humanin analog S14G (HNG) or SHLP2 (n = 6 per group). Vehicle or peptides were given as intraperitoneal (IP) injections twice a day at dose of 2.5 mg/kg/injection for 3 days. Metabolites in plasma samples were comprehensively identified and quantified using UPLC-MS/MS.

**Results:**

HNG and SHLP2 administration significantly altered the concentrations of amino acid and lipid metabolites in plasma. Among all the metabolic pathways, the glutathione and sphingolipid metabolism responded most strongly to the peptide treatment.

**Conclusions:**

The present study indicates that humanin and SHLP2 can lower several markers associated with age-related metabolic disorders. With the previous understanding of the effects of humanin and SHLP2 on cardiovascular function, insulin sensitization, and anti-inflammation, this metabolomic discovery provides a more comprehensive molecular explanation of the mechanism of action for humanin and SHLP2 treatment.

**Electronic supplementary material:**

The online version of this article (10.1007/s11306-019-1549-7) contains supplementary material, which is available to authorized users.

## Introduction

Mitochondria are highly dynamic organelles that control cellular energetics, biosynthesis of macromolecules and stress response. Different from other organelles in the cell, mitochondria are semi-autonomous as they are surrounded by a double membrane and possess their own small genome which encodes proteins essential for oxidative phosphorylation (OXPHOS) complexes. However, mitochondria are not isolated entities, they form highly connected networks called mitochondrial reticulum, and communicate with the nucleus through retrograde signaling. Mitochondrial retrograde signaling allows communication from mitochondria to the nucleus via secondary messengers including Ca^2+^, ATP/ADP, NAD^+^/NADH ratios, and other small molecules (Durieux et al. 2011; Haynes et al. 2010; Zhang et al. 2018). The cell has developed sophisticated pathways to sense and transmit these mitochondrial signals to modulate nuclear gene expression, leading to cellular remodeling of metabolism to accommodate changes in the mitochondria (Kim et al. 2018). With the advances in the understanding of mitochondrial biology and the establishment of the small open-reading frame (sORF) biology, it is now well-accepted that the mitochondrial genome contains not only the information about energy metabolism, but also the molecular instructions for retrograde signaling. In addition to the aforementioned biochemicals, small peptides encoded in the mtDNA have also been added to the repertoire of mitochondrial secondary messengers mediating signaling events. These mitochondrial-derived peptides (MDPs) are secreted into the circulation and exert a plethora of functions through receptor-mediated signaling pathways (Fuku et al. 2015; Gong et al. 2017; Kim et al. 2018; Klein et al. 2013; Qin et al. 2018; Xiao et al. 2017).

Humanin, which was the first MDP discovered, was identified as a neuroprotective peptide using a screen searching for genes that could protect neuronal cells from amyloid-β toxicity (Hashimoto et al. 2001; Hashimoto et al. 2001a; Hashimoto et al. 2001b; Yen et al. 2018). It was later found to be an IGFBP-3 interacting protein and exerts its anti-apoptotic functions through direct binding to IGFBP-3 (Ikonen et al. 2003). Over the past decade, the effects and mechanism of humanin action have been carefully characterized (Harada et al. 2004; Kim et al. 2016; Muzumdar et al. 2009; Nashine et al. 2017; Qin et al. 2018; Yen et al. 2018; Ying et al. 2004). Humanin protects cells against various insults, such as oxidative damage and amyloid toxicity (Chai et al. 2014; Klein et al. 2013; Okada et al. 2017; Paharkova et al. 2015; Romeo et al. 2017; Thummasorn et al. 2016; Thummasorn et al. 2017; Thummasorn et al. 2017). It also suppresses chemotherapy-induced apoptosis in normal blood cells, while sensitizing tumor cells to chemotherapy (Lue et al. 2015). In addition to cytoprotection, humanin administration has several metabolic benefits including enhanced insulin sensitivity and better cardiac function (Muzumdar et al. 2009; Qin et al. 2018; Qin et al. 2018; Zhang et al. 2012). As for the regulation of endogenous humanin, it was found that its levels decreased with age, and that it is suppressed by the growth hormone/insulin-like growth factor-1 axis (GH/IGF-I axis), which is one of the most important pathways in aging (Lee et al. 2014; Mao et al. 2018; Muzumdar et al. 2009).

The discovery and comprehensive study of humanin has built a foundation for more novel MDPs to be identified and characterized. In fact, mitochondrial peptides MOTS-c and small humanin-like peptides (SHLPs) were discovered and reported to have important regulatory effects on metabolism. SHLP 1–6 are circulating peptides of approximately 20 amino acids in length and are encoded within the same 16S rRNA in which humanin is located. SHLP2 exhibits similar effects as humanin, in terms of anti-apoptosis, insulin sensitization and maintenance of glucose homeostasis (Cobb et al. 2016). Furthermore, in vitro SHLP2 treatment also promotes mitochondrial health by increasing oxygen consumption rate (OCR) and ATP generation. Recently, we have reported that SHLP2 plays a key role in the development and racial disparity of prostate cancer, as low levels of SHLP2 are linked with increased prostate cancer in white men (Xiao et al. 2017).

MDPs are encoded within the mitochondrial genome to carry on crucial signaling tasks and are involved in a broad range of metabolic events. So far, most of the studies on MDPs have focused on one disease model or one biological effect at a time. This targeted research approach, although allowing for deep investigation, may nevertheless provide limited information. Humanin and SHLP2 have been shown to enhance metabolic fitness, and exhibit protection against metabolic syndromes such as cardiovascular diseases, type-2 diabetes and cancer. It is intriguing that many of these diseases are associated with obesity, therefore we speculated that humanin and humanin-like peptides may have effects in obesity-induced pathobiology. In this study, we administered the potent humanin analog HNG or SHLP2 in diet-induced obesity (DIO) mice. Blood samples were collected, and plasma was sent for metabolome analysis to obtain a comprehensive understanding of the changes in the metabolite profile and metabolic pathways.

## Methods

### Animals and sample collection

12 week old, male, dietary-induced obesity (DIO) C57BL/6 J mice were purchased from Jackson Laboratory (Bar Harbor, ME, USA). The mice were housed 3 per cage and under standard 12 h light–dark cycle with access to water and high fat (60% fat) rodent food ad libitum (Research Diets #D12492). Mice were randomly assigned to one of three experimental groups (n = 6 per group): a control group receiving daily Intraperitoneal (IP) injection of vehicle (sterilized water); a HNG-treated group receiving twice daily IP injection of 2.5 mg HNG per kg body weight; or a SHLP2-treated group receiving twice daily IP injection of 2.5 mg SHLP2 per kg body weight. Both peptides were synthesized and purchased from Genscript, Piscataway, NJ and reconstituted in water immediately before injection. Mice were euthanized after 3 days of treatment. Blood was collected in blood collection tubes with spray-coated K2EDTA (Becton–Dickinson, USA) and cells were removed by centrifugation at 1500×*g* for 10 minutes at 4 °C. The resulting supernatant (plasma) was transferred and aliquoted into Eppendorf tubes, then immediately stored at − 80 °C. The plasma samples were then shipped on dry ice to Metabolon (NC, USA) for subsequent fractionation, mass-spectrometry and analysis. There were no differences between bodyweight or food intake between groups (Supplemental Fig. 1). All experiments with mice were performed in accordance with the appropriate guidelines and regulations and approved by the University of Southern California Institutional Animal Care and Use Committee (IACUC) under protocol #20787.

### Data analysis

Initial analysis of data was performed by Metabolon (NC, USA) as described previously (Lee et al. 2015). One-way ANOVA (analysis of variance) and Tukey & Dunnett multiple comparisons were conducted to identify biochemicals that differed between control and peptide treatment when comparing the metabolic profiles of plasma samples (ArrayStudio). P values < 0.05 were considered statistically significant. The level of 0.05 is the false positive rate when there is one test. However, for a large number of tests we need to account for false positives. The FDR was estimated using the q-value (ArrayStudio). The additional tests, including hierarchical clustering, Random Forest analysis, principal component analysis (PCA) were conducted by using RStudio version 1.0.143.

### Western blot

Tissue was lysed with RIPA buffer (25 mM TrisHCl pH 7.6, 150 mM NaCl, 1% NP-40, 1% sodium deoxycholate, 0.1% SDS) supplemented with Halt protease and phosphatase inhibitor cocktail (ThermoFisher Scientific). The lysates were homogenized using a sonicator, and the supernatant was collected by centrifugation at 19,000×*g* for 15 minutes at 4 °C. Protein content in the lysates was quantified using the PierceTM BCA Protein Assay Kit (ThermoFisher Scientific). A total of 30 μg protein/well were separated on 8–16% SDS-PAGE gels and blotted onto PVDF membranes (BioRad). Membranes were incubated with primary anti-CPT1A antibody (128568, abcam) and anti-β-actin antibody (A5316, Sigma) at 4 °C overnight, according to the manufacturer’s instructions. After several washes with Tris-buffered saline containing 0.1% Tween-20, membranes were incubated at room temperature for 1 hour with the appropriate HRP-conjugated secondary antibody. Enhanced chemiluminescence was used for detecting specific bands. Membranes were imaged on a Bio-Rad ChemiDoc XRS + imager. Relative band intensities in each condition were quantified using ImageJ, a free software provided by National Institute of Health (Bethesda, Maryland, USA).

### qPCR

Total RNA was isolated from liver tissue using Trizol lysis followed by Zymo extraction according to the manufacture protocol (CAT: R2054). RNA samples (1 ug) were reverse transcribed to cDNA was using iScript cDNA synthesis kit (CAT: 1708890). Ssoadvanced Universal SYBR green supermix was used to amplify cDNA. For relative gene expression analysis, the 2 − ΔΔCT method was used, which measures the fold increase (or decrease) of the target gene in the test sample relative to the calibrator sample and is normalized to the expression of a reference gene. Target genes were normalized to the CT of the reference gene for both the test and calibrator sample (ΔCT). Then, the ΔCT of the test sample was normalized to the ΔCT of the calibrator sample (ΔΔCT). Finally, the expression ratio was calculated by 2 − ΔΔCT and then examined data relative to the control samples. The following primers were used: *Actin* forward (5′CCAACCGCGAGAAGATGA3′) *Actin* reverse (5′TCCATCACGATG CCAGTG3′), *Cpt1A* forward (5′CCGTGAGGAACTCAAACCTATT3′) *Cpt1A* reverse (5′CAGGGATGCGGGAAGTATTG3′), *SphK1* forward (*5′*GGTACGAGCAGGTGACTAATG3′) *SphK1* reverse (*5′*GGACAGACTGAGCACAGAATAG3′), *Spp1* forward (*5′*CCATTGGTGGACCTGATTGA3′) *Spp1* reverse (*5′*GTGTCAAGGGTGAAAGAGAAGA ·3′), *Spt* forward (*5′*GACAAGAAGACCCTGGAAGAAA3′) *Spt* reverse (*5′*CTTCAGGAAGGCGAACAATAGA3′), *Ceramidase* forward (5*′*GTGTCAGAAACCCAAACCAATC3′) *Ceramidase* reverse (*5′*GGACCCAACGGTAACAATCT3′) *Ggt* forward (*S*AACAGAAGGCACTGACGTATC3′) *Ggt* reverse (*5′*CCTGAGACACATCGACAAACT3′), *Gpx*-*1* forward (*5′*CCCGTGCAATCAGTTC -3′) *Gpx*-*1* reverse (*5′*TTCGCACTTCTCAAACAA3′).

### Determining changes in GSH

GSH concentration was measured using the commercially available GSH-Glo™ Glutathione Assay (Promega) following the manufacturer’s instructions. Briefly, 10 mg frozen liver tissues were homogenized in 1 ml PBS containing 2 mM EDTA. The lysates were incubated with the GSH-Glo™ Reagent and Luciferin Detection Reagent. Luminescence of liver tissue lysates from HNG and SHLP2 injected DIO mice were compared to the controls to determine changes in GSH.

## Results

### Multivariate analysis

**Fig. 1:**
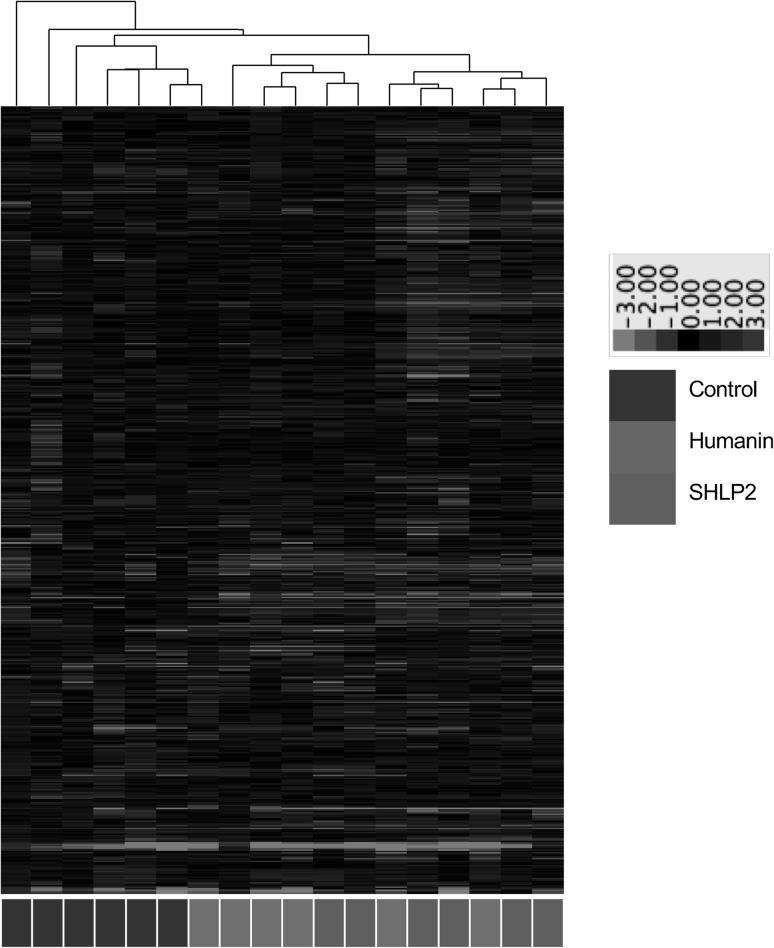
Hierarchical clustering analysis of the metabolomic data. Red color indicates the abundance of metabolite were up-regulated compared to that of the control and green color indicates down-regulation. (See ratio color key)

The present dataset comprises a total of 549 compounds of known identity (named biochemicals). Plasma levels of 52 biochemicals changed significantly (14 rose and 38 fell) in response to HNG treatment, while 77 biochemicals changed significantly (16 rose and 61 fell) in response to SHLP2 treatment. The majority of these metabolites could be categorized into the 4 categories as will be discussed below. Figure 1 is hierarchical clustering analysis demonstrating evidence of peptide-specific effects. Hierarchical clustering is used to compare similarities and differences between metabolite profiles. When using the changes in all 549 biochemicals, the control mice were clustered in one group while the HNG and SHLP2 groups exhibited a more overlapping pattern of changes. As it is known that both humanin and SHLP2 have unique and overlapping signaling mechanisms (Cobb et al. 2016), it is not surprising to see some similarity between the two peptides. Comparing between HNG and SHLP2 treatments, there were only 16 metabolites that were significantly different from each other. These differences are summarized in supplemental Table 1.

**Table 1:** Changes in acylcarnitines and malonate levels when treated with HNG

Metabolite	CID	Pathway	Fold change	*p value*
3-Hydroxybutyrylcarnitine	53481617	Fatty acid metabolism	**0.39**	0.010
Acetylcarnitine	1	Fatty acid metabolism	0.79	0.241
Adipoylcarnitine	71296139	Fatty acid metabolism	**0.54**	0.032
Cis-4-decenoyl carnitine	57357170	Fatty acid metabolism	0.68	0.120
Decanoylcarnitine	10245190	Fatty acid metabolism	0.77	0.206
Hexanoylcarnitine	6426853	Fatty acid metabolism	0.62	0.210
Laurylcarnitine	10427569	Fatty acid metabolism	0.77	0.285
Linoleoylcarnitine	6450015	Fatty acid metabolism	**0.72**	0.025
Myristoleoylcarnitine	129691961	Fatty acid metabolism	0.71	0.088
Myristoylcarnitine	6426854	Fatty acid metabolism	0.73	0.156
Octanoylcarnitine	123701	Fatty acid metabolism	0.86	0.556
Oleoylcarnitine	6441392	Fatty acid metabolism	**0.71**	0.009
Palmitoleoylcarnitine	71464547	Fatty acid metabolism	**0.66**	0.044
Palmitoylcarnitine	461	Fatty acid metabolism	0.82	0.113
Stearoylcarnitine	6426855	Fatty acid metabolism	0.93	0.203
Malonate	867	Fatty acid synthesis	**0.74**	0.049

Bold indicates significant difference (*p *≤ 0.05), metabolite ratio of < 1.00

**Fig. 2:**
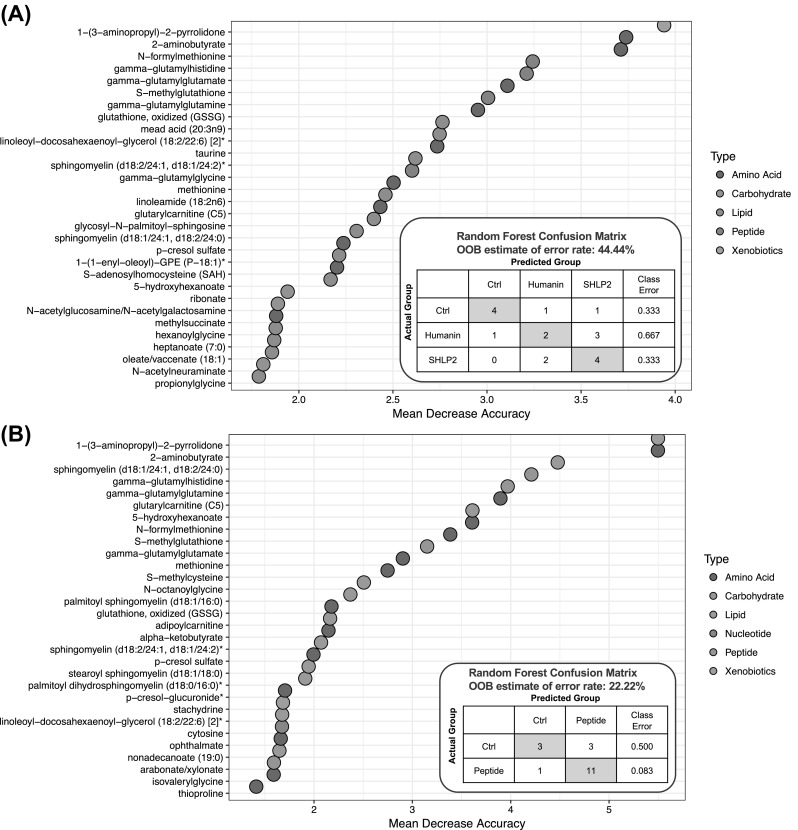
Random Forest classification using named metabolites in plasma of control compared to plasma of Humanin and SHLP2 treated mice. The dot chart displays the top 30 Mean Decrease Accuracy values calculated for **a** the comparison of all three sample groups. A higher Mean Decrease Accuracy value indicates a greater group differentiating contribution. Amino acid and lipid metabolites were highly represented in the top 30. **b** The comparison between control group and peptide treatment group (Humanin and SHLP2)

**Fig. 3:**
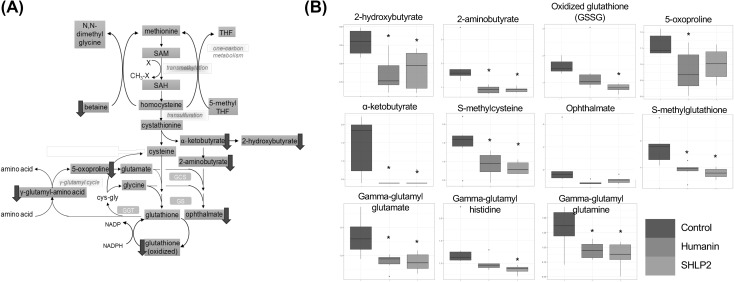
The metabolite profile of the transmethylation, transulfuration and gamma-glutamyl cycle. **a** The schematic diagram of the pathway. Yellow box indicates metabolite not measured in the metabolomic discovery, green box indicates metabolite measured but did not change in response to peptide treatment, blue box indicates metabolite that was down-regulated compared to control. **b** Box plots of selected biochemicals. Values are normalized raw area counts rescaled to set the median equal to 1. * indicates p < .05 compared to control

Random Forest (RF) analysis attempts to bin individual samples in groups based on their metabolite similarities and differences. Random Forest also defines which metabolites contribute most strongly to the grouping process. Figure 2a presents Random Forest results for the control and peptide-treated plasmas. RF was able to properly bin 4 of 6 control samples (predictive accuracy 67%), which is an indication that control sample profiles were unique from the peptide-treated samples. The top 3 differentiating metabolites were 1-(3-aminopropyl)2-pyrrolidone (CID 82111), 2-aminobutyrate (CID 439691), and N-formylmethionine (CID 911). Figure 2a also displays a plot of the top 30 Mean Decrease Accuracy values calculated for the comparison of all five sample groups. A higher Mean Decrease Accuracy value indicates a greater group differentiating contribution. A clear distinction between HNG and SHLP2 treatment groups was not observed and this could be due to a number of reasons. Firstly, HNG and SHLP2 are known to activate common pathways such as the ERK signaling pathway, although at a different kinetic rate. Secondly, the differences in the peptide treatment may not have been noticeable at the plasma level and an examination of the tissue specific effects could further distinguish the two peptide treatments. In fact, our supplementary data figure S2 suggests that in the liver, there are distinct effects on transcript levels such as the case of *Sphk* and *Ggt*. Thirdly, because the experiment was not setup to distinguish between the two different peptides, it could have been underpowered to observe the subtle differences between peptide groups. Since we did not observe a clear distinction between HNG and SHLP2 treatment, we performed a second RF test taking HNG and SHLP2 as a single “peptide treated” group. Figure 2b demonstrated that RF properly bin 11 of 12 treatment samples. The metabolite with the highest mean-decrease-accuracy value were 2-aminobutyrate (CID 439691) and 1-(3-aminopropyl)-2-pyrrolidone (CID 82111). The former is a metabolite generated in the process of transsulfuration to generate cysteine. Statistical comparison of 2-aminobutyrate levels in control versus each of the peptides generated the lowest p-values among all the measured metabolites. Amino acid and lipid metabolites were highly represented in the top 30. Furthermore, we found that the metabolites from sphingolipid metabolism, glutathione and gamma glutamyl transferase (GGT) metabolism and methionine, cysteine, SAM and taurine metabolism were overrepresented, which suggests that these biological pathways are most influenced by HNG and SHLP2 (Fig. 3a). PCA permits visualization of how individual samples, within a group, cluster with respect to their “principle components” calculated from the relative changes in the selected metabolites. As such, this analysis tool aids in determining if the plasma from peptide-treated mice can be differentiated from control, or from each other, based on differences in their overall metabolite signature. Comparing control to all peptide-treated samples, there appears to be some degree of segregation on the x-axis (component 1). There was substantial variability across all groups, which may reflect animal to animal variability in response to peptide.

### Changes in metabolite profile with HNG or SHLP2 treatment

Overall, we can group the altered metabolites based on their biological pathways into 4 categories:

1. *The methionine cycle and glutathione metabolism* The metabolites alpha-ketobutyrate (CID 58), 2-hydroxybutyrate (CID 440864) (an isobar of 2-hydroxyisobutyrate where 2-hydroxybutyrate predominates) and 2-aminobutyrate (CID 439691) (top metabolite in RF analysis above) significantly decreased in response to peptide administration, which may be indicative of reduced cysteine synthesis from cystathionine (p < .05). However, plasma cysteine (CID 5862) levels were relatively consistent between groups, as were methionine (CID 6137) and cystathionine (CID 439258). The generation of cysteine from methionine through the transsulfuration pathway occurs primarily in liver and kidneys. It is possible that the secretion of methionine, cystathionine and cysteine into plasma is not affected by subtle changes in the syntheses. Betaine (CID 247), which is utilized to convert homocysteine back to methionine, was lower in all peptide-treated plasma, possibly consistent with greater recycling of homocysteine and less homocysteine conversion to cystathionine to support cysteine and glutathione synthesis. Oxidized glutathione (CID 65359) was lower to a statistically significant degree (p < .05) in SHLP2 treated mice but not in HNG treated mice, relative to control (Fig. 3). Checking the mRNA levels of glutathione related enzymes such as *Ggt and Gpx*, we find a significant decrease in HNG treated mice for *Ggt* and a significant increase in *Gpx* transcript for both treatments (Supplemental Figure S2).
2. *Gamma-glutamyl-amino acid* the plasma levels of several gamma-glutamyl amino acids were significantly lowered, including gamma-glutamyl glutamate (CID 92865), gamma-glutamyl glutamine (CID 126296) and gamma-glutamyl histidine (for SHLP2 only) (CID 7017195). In addition, the concentration of the downstream metabolite 5-oxoproline (CID 7405) also decreased significantly for the HNG group and trended downwards for the SHLP2 group. Gamma-glutamyl amino acids are made through adding a gamma-glutamyl functional group from molecules such as glutathione to an amino acid and this reaction is catalyzed by the enzyme gamma-glutamyl transferase (GGT). This result suggests that HNG and SHLP2 suppress gamma-glutamyl-amino acid cycle through (1) down-regulation of GGT activity; (2) reducing availability of glutathione. However, the latter hypothesis is less likely, since the levels of cysteine, glutamate, and glycine were not affected (Fig. 4).

**Fig. 4:**
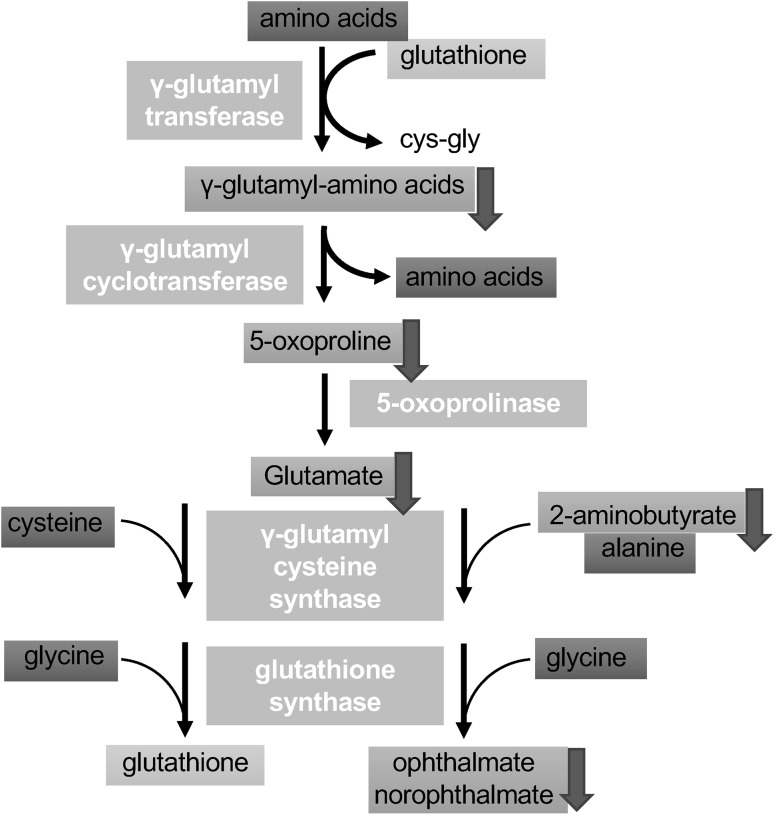
The metabolite profile of the gamma-glutamyl cycle. Yellow box indicates metabolite not measured in the metabolomic discovery, green box indicates metabolite measured but did not change in response to peptide treatment, blue box indicates metabolite that was down-regulated compared to control
3. *Sphingolipid metabolism* sphingolipids play significant roles in cell membranes and provide many bioactive metabolites that regulate cell function. From this metabolome study, we observed consistent modulation induced by these MDPs, which was a reduction in multiple components of the sphingolipids pathway. Sphinganine (CID 3126), which is a precursor to ceramide, decreased significantly in HNG and SHLP2 treated plasma, relative to control. This may suggest a reduction in synthesis in tissues. Furthermore, two glycosylated ceramides, glycosyl-N-palmitoyl-sphingosine and glycosyl-N-steroylsphingosine, displayed strong decreases with both peptides. Multiple types of sphingomyelin also exhibited significant reduction. Sphingomyelin is synthesized from the enzymatic transfer of a phosphocholine to a ceramide with diacylglycerol being produced as a byproduct. We also observed a trend of decreases in plasma diacylglycerol in response to HNG/SHLP2 treatment, which suggests that the synthesis of sphingomyelin from ceramide was impeded (Fig. 5). Checking liver mRNA levels of sphingolipid related enzymes found no significant changes although there was a trend for a decrease sphingosine kinase 1 gene in the HNG treated group (Supplemental Figure S2).

**Fig. 5:**
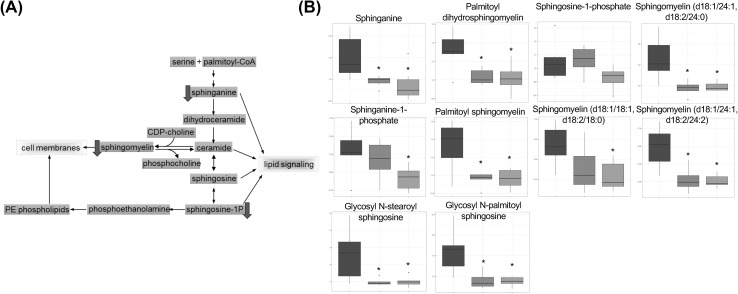
The metabolite profile of the sphingolipid metabolism. **a** The schematic diagram of the pathway. Yellow box indicates metabolite not measured in the metabolomic discovery, green box indicates metabolite measured but did not change in response to peptide treatment, blue box indicates metabolite that was down-regulated compared to control. **b** Box plots of selected biochemicals. Values are normalized raw area counts rescaled to set the median equal to 1. * Indicates p < .05 compared to control
4. *Acylcarnitine metabolism* We found that several long-chain acylcarnitines were lower in HNG-treated mice (Table 1) but not in SHLP2-treated mice. Carnitine is conjugated to fatty acids, by the enzyme carnitine palmitoyl transferase (CPT), to facilitate their transport across the mitochondrial membrane. The activity of CPT is considered a rate-limiting step in fatty acid β-oxidation. Elevated levels of acylcarnitines in cells can indicate increased levels of fatty acid β -oxidation, whereas higher concentration of acylcarnitines in plasma may suggest inefficient fatty acid breakdown, with subsequent export of excess acylcarnitines from cells ending up in the blood stream. Under these circumstances, the HNG-dependent decrease in acylcarnitines might be consistent with a higher efficiency of mitochondrial oxidative phosphorylation shown in other studies. Checking both mRNA levels and protein levels in the liver of treated mice (Supplemental Figures S2 and S3), we found no change in the message level, but a significant increase in HNG treated mice.

## Discussion

### Key intermediates in the γ-glutamyl cycle were reduced in plasma from peptide treated mice

The γ-glutamyl cycle is an important process in the cell to maintain a normal redox state; it generates glutathione to scavenge ROS, and thereby avoiding detrimental oxidative stress (Pompella et al. 2007). The thiol group of cysteine in glutathione is able to donate electron to other molecules, such as reactive oxygen species to neutralize them. By contributing an electron, glutathione can be converted to its oxidized form, GSSG. Therefore, the ratio of reduced glutathione to oxidized glutathione within cells is often used as a measure of cellular oxidative stress (Griffith et al. 1979). In our metabolomic study, the levels of glutathione in the plasma are not available, because the non-targeted approach we utilized is not an ideal way to measure labile biochemicals that are very rapidly oxidized in blood. Although the ratio of glutathione: GSSG cannot be calculated, the reduction of GSSG in plasma still implicated reduced oxidative stress in tissues (Harris and Hansen 2012).

2-Hydroxybutyrate is released as a byproduct when cystathionine is cleaved to cysteine that is incorporated into glutathione, which can be further catalyzed to form 2-aminobutyrate. When there is increased oxidative burden and glutathione consumption, ophthalmate can be synthesized from glutamate and 2-aminobutyrate as a tripeptide analogue of glutathione (Fig. 5). For this reason, the elevation of ophthalmate and intermediates in this pathway is considered a potential biomarker for glutathione depletion following oxidative stress (Soga et al. 2006). It was also reported that ophthalmate, 2-aminobutyrate and 2-hydroxybutyrate increased significantly in various cancer types including breast cancer, ovarian cancer, and oral cancer (Fong et al. 2011; Tan et al. 2013). Moreover, the elevated levels of 5-oxoproline, an amino acid derivative in the glutathione cycle, is strongly correlated with increased glutathione consumption in colorectal cancer (Qiu et al. 2014). These aforementioned metabolites were reduced in plasma from HNG and SHLP2 treated DIO mice, which is indicative of lower ROS levels in tissues and a decrease in demand for glutathione. This metabolic signature also agrees with previous studies demonstrating the anti-ROS benefits of humanin and SHLP2 (Paharkova et al. 2015); (Cobb et al. 2016; Klein et al. 2013; Matsunaga et al. 2016; Sreekumar et al. 2016; Thummasorn et al. 2017). Furthermore, suppression of the key intermediates in glutathione synthesis pathway may help explain why high SHLP2 levels are associated with lower risk of prostate cancer, as oxidative stress is involved in all aspects of tumor development (Xiao et al. 2017).

### Peptide treatment decreased sphingolipids

**Fig. 6:**
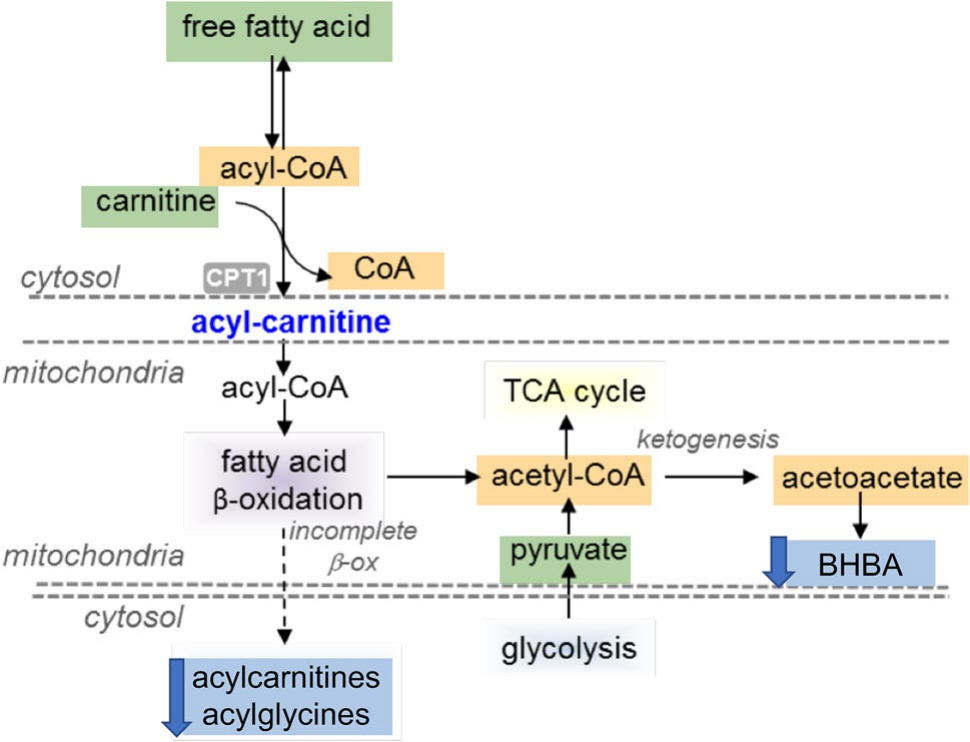
The metabolite profile of the carnitine metabolism pathway. The schematic diagram of the pathway. Yellow box indicates metabolite not measured in the metabolomic discovery, green box indicates metabolite measured but did not change in response to peptide treatment, blue box indicates metabolite that was down-regulated compared to control

Sphingolipids are important structural components of cell membranes and play essential roles in cell signaling. Sphingolipids are primary components in lipid rafts, which are stabilized areas on the plasma membrane that are enriched in sphingomyelin and cholesterol. The lipid rafts stabilize the membrane, serve as attachment points for proteins and provide structural scaffolding (Gault et al. 2010). With their structural and signaling roles in cell and plasma membranes, sphingolipids are involved in insulin-action and inflammation (Kang et al. 2013; Li et al. 2011). In addition, the sphingolipids ceramide and sphingosine 1-phosphate (S1P) are mediators of apoptotic signaling (Ponnusamy et al. 2010). Several reports have linked sphingolipids to normal and abnormal aging and their levels are elevated in diabetes and obesity (Samad et al. 2006; Shah et al. 2008). Sphingomyelin is one of the most abundant sphingolipid and acts as both membrane component and pool for rapid ceramide generation. Several studies showed that high-fat-diet (HFD) increased sphingomyelin levels in liver, adipose tissue and plasma (Russo et al. 2013). High concentration of sphingomyelin has also been correlated with coronary artery disease in obese patients. S1P, a potent bioactive sphingolipid, was found to be significantly higher in liver, skeletal muscle, and plasma in response to several experimental models of HFD. While S1P in liver and skeletal muscle was unchanged by HFD or palmitate in some studies, S1P in plasma was consistently elevated, which suggests that circulating S1P may mediate some of the systemic effects induced by HFD and obesity (Choi and Snider 2015). Furthermore, there is a positive correlation between plasma S1P and body fat percentage, body mass index, waist circumference, and fasting plasma insulin in obese humans (Kowalski et al. 2013) (Fig. 6).

The ability of humanin to suppress ceramide synthesis has been demonstrated in in vitro models (Bachar et al. 2010). However, the two ceramides (N-palmitoyl-sphingosine (C16:0) and N-stearoyl-sphingosine (C18:0)) measured in current study did not exhibit significant decrease in plasma. This result does not exclude the possibility that synthesis of C16:0 and C18:0 ceramides were indeed suppressed by HNG, because circulating ceramide levels might not promptly reflect tissue levels. Moreover, the reduction in S1P and sphingomyelin further suggests that the peptide treatments restored the HFD-induced dysregulation of sphingolipids and reduced sphingolipid levels might partially explain their anti-apoptosis and insulin-sensitizing effects. It has also been reported that AMPK activation inhibits ceramide synthesis at the serine palmitoyl transferase step (Erickson et al. 2012). Consistent with this notion, MDPs have been shown to activate AMPK in vivo. The role of sphingolipids in obesity and metabolic aging is still emerging, our findings of reduced circulating levels of sphingolipids in HNG and SHLP2 treated mice provide a new putative biochemical explanation for their importance in maintaining metabolic fitness during aging.

### Similarities between HNG and SHLP2

Currently, we know that HNG and SHLP2 are both made from the mitochondrial 16S rRNA and have some common biological functions. For example, they both activate ERK and STAT3 signaling pathways, and exhibit insulin sensitization as well as anti-apoptosis effects (Cobb et al. 2016). As was seen in the PCA analysis and hierarchical clustering, clustering of control samples is observed, however, the clustering and separation between HNG and SHLP2 treated samples are limited. Limited hierarchical clustering or PCA group separations can be an indication that the overall metabolite profiles are somewhat similar, but this does not exclude the possibility that individual metabolite levels may be significantly different between groups.

Humanin is known to exert its diverse functions through extracellular membrane receptors and intracellular binding partners. Binding of humanin to the CNTF, WSX1 and GP130 trimeric receptor leads to STAT3 phosphorylation and activation of downstream pathways and it has a central effect (Gong et al. 2015; Kim et al. 2016). As for SHLP2, it has been reported that SHLP2 also activated STAT3 pathways in a time-dependent manner but with different kinetics than those induced by humanin, however the mechanism prior to STAT3 action remains unknown (Cobb et al. 2016). Through this study, we established more metabolic similarities between HNG and SHLP2, and these metabolic similarities may be the result of one or two common pathways. Alternatively, it has recently been shown that both HNG and SHLP2 exhibit chaperone like activity and this common feature may also contribute to the overlapping effects (Okada et al. 2017). Other studies have also linked both humanin and SHLP2 to protection and eye disease (Nashine et al. 2017, 2018; Okada et al. 2017; Sreekumar et al. 2016; Terluk et al. 2015). As there are still many more MDPs (Cobb et al. 2016; Du et al. 2018; Kim et al. 2018; Nashine et al. 2018; Xiao et al. 2017; Zhai et al. 2017), it is likely that some will have overlapping metabolic similarities as well as distinct ones as was the case for these two peptides.

## Conclusions

PCA and Random Forest analysis indicated that the plasma metabolite profiles of peptide-treated mice differed from controls and the reduction of major intermediates in the glutathione cycle and sphingolipid pathways. The observation of peptide-dependent reductions in sphingolipids is very intriguing, given their role in lipid signaling and a potential connection of ceramides to insulin resistance and apoptosis. Peptide effects on glutathione metabolism were also meaningful, and more detailed examination of the relevant pathway in specific tissues may offer greater insight. In addition, future metabolic characterization of tissues, such as liver and muscle, in peptide-treated mice will be very instructive and shed light on the complex biochemical events triggered by MDPs.

## Electronic supplementary material

Below is the link to the electronic supplementary material.

Supplementary material 1 (PDF 44 kb)

Supplementary material 2 (PDF 149 kb)

Supplementary material 3 (PDF 510 kb)

Supplementary material 4 (DOCX 11 kb)

## References

[CR1] Bachar AR, Scheffer L, Schroeder AS, Nakamura HK, Cobb LJ, Oh YK (2010). Humanin is expressed in human vascular walls and has a cytoprotective effect against oxidized LDL-induced oxidative stress. Cardiovascular Research.

[CR2] Chai G-S, Duan D-X, Ma R-H, Shen J-Y, Li H-L, Ma Z-W (2014). Humanin attenuates Alzheimer-like cognitive deficits and pathological changes induced by amyloid β-peptide in rats. Neuroscience bulletin.

[CR3] Choi S, Snider AJ (2015). Sphingolipids in high fat diet and obesity-related diseases. Mediators of Inflammation.

[CR4] Cobb LJ, Lee C, Xiao J, Yen K, Wong RG, Nakamura HK (2016). Naturally occurring mitochondrial-derived peptides are age-dependent regulators of apoptosis, insulin sensitivity, and inflammatory markers. Aging.

[CR5] Du C, Zhang C, Wu W, Liang Y, Pediatric AW (2018). Circulating MOTS-c levels are decreased in obese male children and adolescents and associated with insulin resistance. Advanced Science.

[CR6] Durieux J, Wolff S, Dillin A (2011). The cell-non-autonomous nature of electron transport chain-mediated longevity. Cell.

[CR7] Erickson KA, Smith ME, Anthonymuthu TS, Evanson MJ (2012). AICAR inhibits ceramide biosynthesis in skeletal muscle. Diabetol Metab Syndr.

[CR8] Fong MY, McDunn J, Kakar SS (2011). Identification of metabolites in the normal ovary and their transformation in primary and metastatic ovarian cancer. PLoS ONE.

[CR9] Fuku N, Pareja-Galeano H, Zempo H, Alis R, Arai Y, Lucia A, Hirose N (2015). The mitochondrial-derived peptide MOTS-c: a player in exceptional longevity?. Aging Cell.

[CR10] Gault Christopher R., Obeid Lina M., Hannun Yusuf A. (2010). An Overview of Sphingolipid Metabolism: From Synthesis to Breakdown. Advances in Experimental Medicine and Biology.

[CR11] Gong Z, Su K, Cui L, Tas E, Zhang T, Dong HH (2015). Central effects of humanin on hepatic triglyceride secretion. American journal of physiology Endocrinology and metabolism.

[CR12] Gong Z, Tasset I, Diaz A, Anguiano J, Tas E, Cui L (2017). Humanin is an endogenous activator of chaperone-mediated autophagy. The Journal of cell biology..

[CR13] Griffith OW, Bridges RJ, Meister A (1979). Transport of gamma-glutamyl amino acids: role of glutathione and gamma-glutamyl transpeptidase. Proceedings of the National academy of Sciences of the United States of America.

[CR14] Harada M, Habata Y, Hosoya M, Nishi K, Fujii R, Kobayashi M, Hinuma S (2004). N-Formylated humanin activates both formyl peptide receptor-like 1 and 2. Biochemical and Biophysical Research Communications.

[CR15] Harris Craig, Hansen Jason M. (2012). Oxidative Stress, Thiols, and Redox Profiles. Methods in Molecular Biology.

[CR16] Hashimoto Y, Ito Y, Niikura T, Shao Z, Hata M, Oyama F, Nishimoto I (2001). Mechanisms of neuroprotection by a novel rescue factor humanin from Swedish mutant amyloid precursor protein. Biochemical and Biophysical Research Communications.

[CR17] Hashimoto Y, Niikura T, Ito Y, Sudo H, Hata M, Arakawa E (2001). Detailed characterization of neuroprotection by a rescue factor humanin against various Alzheimer’s disease-relevant insults. The Journal of neuroscience: the official journal of the Society for Neuroscience.

[CR18] Hashimoto Y, Niikura T, Tajima H, Yasukawa T, Sudo H, Ito Y (2001). A rescue factor abolishing neuronal cell death by a wide spectrum of familial Alzheimer’s disease genes and Abeta. Proceedings of the National academy of Sciences of the United States of America.

[CR19] Haynes CM, Yang Y, Blais SP, Neubert TA, Ron D (2010). The matrix peptide exporter HAF-1 signals a mitochondrial UPR by activating the transcription factor ZC376.7 in *C. elegans*. Molecular Cell.

[CR20] Ikonen M, Liu B, Hashimoto Y, Ma L, Lee K-W, Niikura T (2003). Interaction between the Alzheimer’s survival peptide humanin and insulin-like growth factor-binding protein 3 regulates cell survival and apoptosis. Proceedings of the National academy of Sciences of the United States of America.

[CR21] Kang S-C, Kim B-R, Lee S-Y, Park T-S (2013). Sphingolipid metabolism and obesity-induced inflammation. Frontiers in endocrinology.

[CR22] Kim S-J, Guerrero N, Wassef G, Xiao J, Mehta HH, Cohen P, Yen K (2016). The mitochondrial-derived peptide humanin activates the ERK1/2, AKT, and STAT3 signaling pathways and has age-dependent signaling differences in the hippocampus. Oncotarget.

[CR23] Kim S-J, Mehta HH, Wan J, Kuehnemann C, Chen J, Hu J-F (2018). Mitochondrial peptides modulate mitochondrial function during cellular senescence. Aging.

[CR24] Kim KH, Son JM, Benayoun BA, Lee C (2018). The mitochondrial-encoded peptide MOTS-c translocates to the nucleus to regulate nuclear gene expression in response to metabolic stress. Cell Metabolism.

[CR25] Klein LE, Cui L, Gong Z, Su K, Muzumdar R (2013). A humanin analog decreases oxidative stress and preserves mitochondrial integrity in cardiac myoblasts. Biochemical and Biophysical Research Communications.

[CR26] Kowalski GM, Carey AL, Selathurai A, Kingwell BA, Bruce CR (2013). Plasma Sphingosine-1-Phosphate Is Elevated in Obesity. PLoS ONE.

[CR27] Lee C, Wan J, Miyazaki B, Fang Y, Guevara-Aguirre J, Yen K (2014). IGF-I regulates the age-dependent signaling peptide humanin. Aging Cell.

[CR28] Lee C, Zeng J, Drew BG, Sallam T, Martin-Montalvo A, Wan J (2015). The mitochondrial-derived peptide MOTS-c promotes metabolic homeostasis and reduces obesity and insulin resistance. Cell Metabolism.

[CR29] Li Z, Zhang H, Liu J, Liang C-P, Li Y, Li Y (2011). Reducing plasma membrane sphingomyelin increases insulin sensitivity. Molecular and Cellular Biology.

[CR30] Lue Y, Swerdloff R, Wan J, Xiao J, French S, Atienza V (2015). The potent humanin analogue (HNG) protects germ cells and leucocytes while enhancing chemotherapy-induced suppression of cancer metastases in male mice. Endocrinology.

[CR31] Mao K, Quipildor GF, Tabrizian T, Novaj A, Guan F, Walters RO (2018). Late-life targeting of the IGF-1 receptor improves healthspan and lifespan in female mice. Nature communications.

[CR32] Matsunaga D, Sreekumar PG, Ishikawa K, Terasaki H, Barron E, Cohen P (2016). Humanin Protects RPE Cells from endoplasmic reticulum stress-induced apoptosis by upregulation of mitochondrial glutathione. PLoS ONE.

[CR33] Muzumdar RH, Muzumdar R, Huffman DM, Huffman D, Atzmon G, Atzmon G (2009). Humanin: a novel central regulator of peripheral insulin action. PLoS ONE.

[CR34] Nashine S, Cohen P, Chwa M, Lu S, Nesburn AB, Kuppermann BD, Kenney MC (2017). Humanin G (HNG) protects age-related macular degeneration (AMD) transmitochondrial ARPE-19 cybrids from mitochondrial and cellular damage. Cell death & disease.

[CR35] Nashine S, Cohen P, Nesburn AB, Kuppermann BD, Kenney MC (2018). Characterizing the protective effects of SHLP2, a mitochondrial-derived peptide, in macular degeneration. Scientific reports.

[CR36] Okada AK, Teranishi K, Lobo F, Isas JM, Xiao J, Yen K (2017). The mitochondrial-derived peptides, HumaninS14G and small humanin-like peptide 2. Exhibit Chaperone-like Activity. Scientific reports.

[CR37] Paharkova V, Alvarez G, Nakamura H, Cohen P, Lee K-W (2015). Rat humanin is encoded and translated in mitochondria and is localized to the mitochondrial compartment where it regulates ROS production. Molecular and Cellular Endocrinology.

[CR38] Pompella A, Corti A, Paolicchi A, Giommarelli C, Zunino F (2007). γ-Glutamyltransferase, redox regulation and cancer drug resistance. Current Opinion in Pharmacology.

[CR39] Ponnusamy S, Meyers-Needham M, Senkal CE, Saddoughi SA, Sentelle D, Selvam SP (2010). Sphingolipids and cancer: ceramide and sphingosine-1-phosphate in the regulation of cell death and drug resistance. Future oncology.

[CR40] Qin Qing, Delrio S, Wan J, Jay Widmer R, Cohen P, Lerman LO, Lerman A (2018). Downregulation of circulating MOTS-c levels in patients with coronary endothelial dysfunction. International Journal of Cardiology.

[CR41] Qin Qingqing, Jin J, He F, Zheng Y, Li T, Zhang Y, He J (2018). Humanin promotes mitochondrial biogenesis in pancreatic MIN6 β-cells. Biochemical and Biophysical Research Communications.

[CR42] Qin Qing, Mehta H, Yen K, Navarrete G, Brandhorst S, Wan J (2018). Chronic treatment with the mitochondrial peptide humanin prevents age-related myocardial fibrosis in mice. Am J Physiol Heart Circ Physiol.

[CR43] Qiu Y, Cai G, Zhou B, Li D, Zhao A, Xie G (2014). A distinct metabolic signature of human colorectal cancer with prognostic potential. Clinical Cancer Research.

[CR44] Romeo M, Stravalaci M, Beeg M, Rossi A, Fiordaliso F, Corbelli A (2017). Humanin specifically interacts with amyloid-β oligomers and counteracts their in vivo toxicity. Journal of Alzheimer’s Disease.

[CR45] Russo S. B., Ross J. S., Cowart L. A. (2013). Sphingolipids in Obesity, Type 2 Diabetes, and Metabolic Disease. Sphingolipids in Disease.

[CR46] Samad F, Hester KD, Yang G, Hannun YA, Bielawski J (2006). Altered adipose and plasma sphingolipid metabolism in obesity: a potential mechanism for cardiovascular and metabolic risk. Diabetes.

[CR47] Shah C, Yang G, Lee I, Bielawski J, Hannun YA, Samad F (2008). Protection from high fat diet-induced increase in ceramide in mice lacking plasminogen activator inhibitor 1. Journal of Biological Chemistry.

[CR48] Soga T, Baran R, Suematsu M, Ueno Y, Ikeda S, Sakurakawa T (2006). Differential metabolomics reveals ophthalmic acid as an oxidative stress biomarker indicating hepatic glutathione consumption. Journal of Biological Chemistry.

[CR49] Sreekumar PG, Ishikawa K, Spee C, Mehta HH, Wan J, Yen K (2016). The mitochondrial-derived peptide humanin protects RPE cells from oxidative stress, senescence, and mitochondrial dysfunction. Investigative Ophthalmology & Visual Science.

[CR50] Tan B, Qiu Y, Zou X, Chen T, Xie G, Cheng Y (2013). Metabonomics identifies serum metabolite markers of colorectal cancer. Journal of Proteome Research.

[CR51] Terluk MR, Kapphahn RJ, Soukup LM, Gong H, Gallardo C, Montezuma SR, Ferrington DA (2015). Investigating mitochondria as a target for treating age-related macular degeneration. The Journal of neuroscience: the official journal of the society for neuroscience.

[CR52] Thummasorn S, Apaijai N, Kerdphoo S, Shinlapawittayatorn K, Chattipakorn SC, Chattipakorn N (2016). Humanin exerts cardioprotection against cardiac ischemia/reperfusion injury through attenuation of mitochondrial dysfunction. Cardiovascular Therapeutics.

[CR53] Thummasorn S, Shinlapawittayatorn K, Chattipakorn SC, Chattipakorn N (2017). High dose humanin analogue applied during ischemia exerts cardioprotection against ischemia/reperfusion injury by reducing mitochondrial dysfunction. Cardiovascular therapeutics.

[CR54] Thummasorn S, Shinlapawittayatorn K, Khamseekaew J, Jaiwongkam T, Chattipakorn SC, Chattipakorn N (2017). Humanin directly protects cardiac mitochondria against dysfunction initiated by oxidative stress by decreasing complex I activity. Mitochondrion.

[CR55] Xiao J, Howard L, Wan J, Wiggins E, Vidal A, Cohen P, Freedland SJ (2017). Low circulating levels of the mitochondrial-peptide hormone SHLP2: novel biomarker for prostate cancer risk. Oncotarget.

[CR56] Yen K, Wan J, Mehta HH, Miller B, Christensen A, Levine ME (2018). Humanin prevents age-related cognitive decline in mice and is associated with improved cognitive age in humans. Scientific reports.

[CR57] Ying G, Iribarren P, Zhou Y, Gong W, Zhang N, Yu Z-X (2004). Humanin, a newly identified neuroprotective factor, uses the G protein-coupled formylpeptide receptor-like-1 as a functional receptor. Journal of Immunology.

[CR58] Zhai D, Ye Z, Jiang Y, Xu C, Ruan B, Yang Y (2017). MOTS-c peptide increases survival and decreases bacterial load in mice infected with MRSA. Molecular Immunology.

[CR59] Zhang X, Urbieta-Caceres VH, Eirin A, Bell CC, Crane JA, Tang H (2012). Humanin prevents intra-renal microvascular remodeling and inflammation in hypercholesterolemic ApoE deficient mice. Life Sciences.

[CR60] Zhang Q, Wu X, Chen P, Liu L, Xin N, Tian Y, Dillin A (2018). The mitochondrial unfolded protein response is mediated cell-non-autonomously by retromer-dependent wnt signaling. Cell.

