# Supplementary figures and images for: Metabolomic profile of diet-induced obesity mice in response to humanin and small humanin-like peptide 2 treatment
Source: Metabolomics. 2019 Jun 6;15(6):88. doi: 10.1007/s11306-019-1549-7 (PMC6554247; doi:10.1007/s11306-019-1549-7)

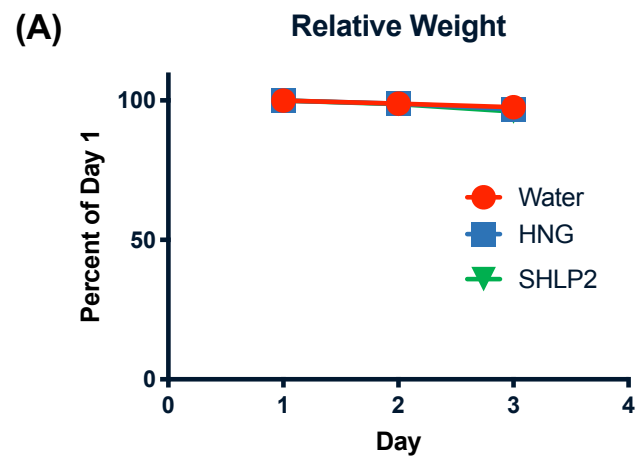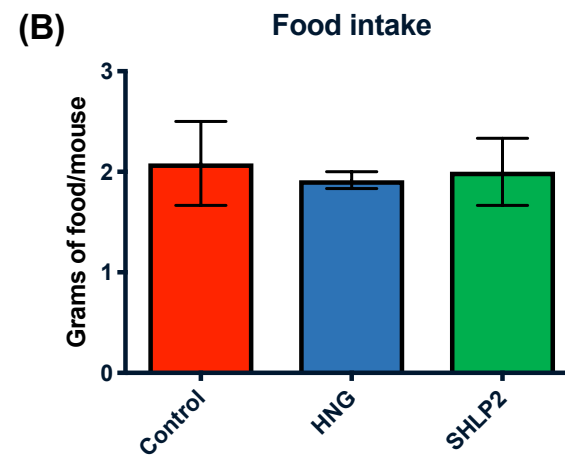

Supplement: Supplementary file 1 — Supplementary material 1 (PDF 44 kb) [file 11306_2019_1549_MOESM1_ESM.pdf]

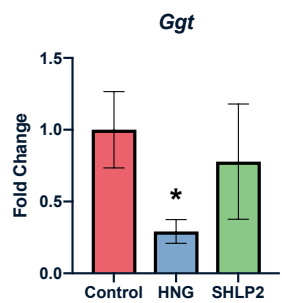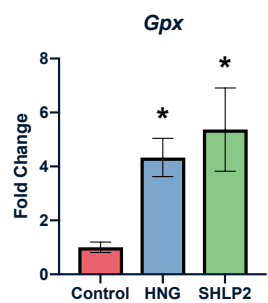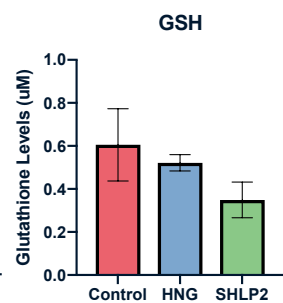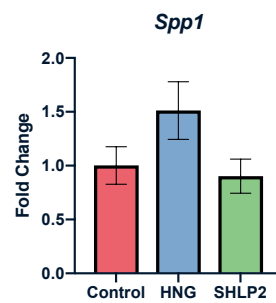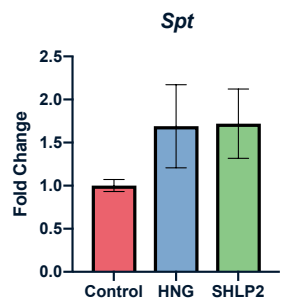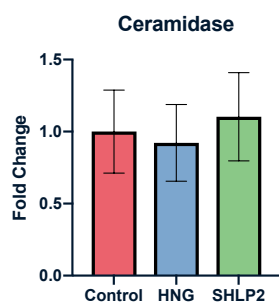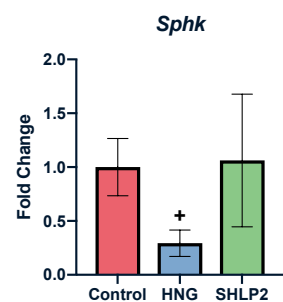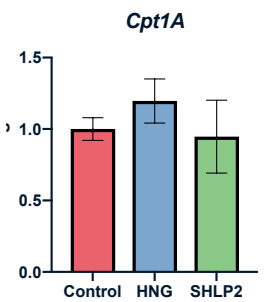

Supplement: Supplementary file 2 — Supplementary material 2 (PDF 149 kb) [file 11306_2019_1549_MOESM2_ESM.pdf]

A.

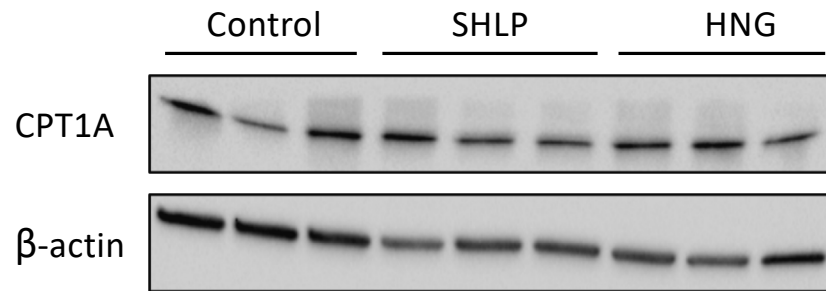

B.

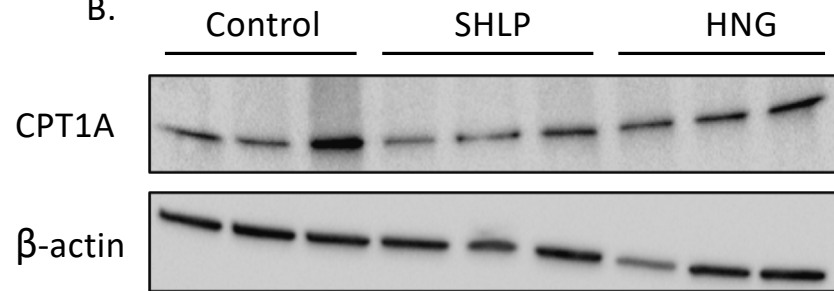

C.

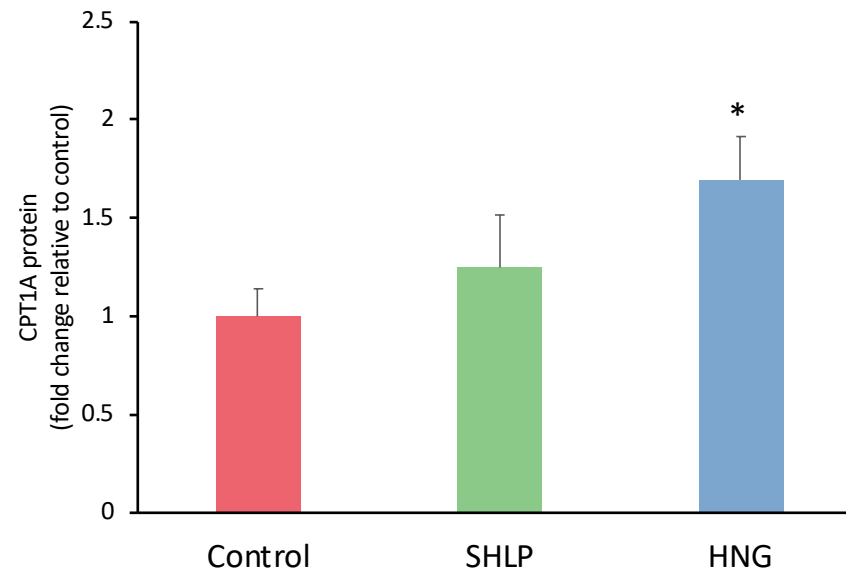

Supplement: Supplementary file 3 — Supplementary material 3 (PDF 510 kb) [file 11306_2019_1549_MOESM3_ESM.pdf]
