# Supplementary material for: Metabolomic profile of diet-induced obesity mice in response to humanin and small humanin-like peptide 2 treatment
Source: Metabolomics. 2019 Jun 6;15(6):88. doi: 10.1007/s11306-019-1549-7 (PMC6554247; doi:10.1007/s11306-019-1549-7)
Supplement: Supplementary file 4 — Supplementary material 4 (DOCX 11 kb) [file 11306_2019_1549_MOESM4_ESM.docx]

| Metabolite | CID | Fold Change |
| --- | --- | --- |
| serine | 1648 | 0.82 |
| thyroxine | 2761 | 0.68 |
| N-acetylkynurenine (2) | 48757 | 1.38 |
| methionine | 1302 | 0.83 |
| 2-oxoarginine* | 55072 | 0.67 |
| glutathione, oxidized (GSSG) | 27727 | 0.59 |
| gamma-glutamylglycine | 33949 | 0.79 |
| glucose | 20488 | 0.81 |
| ribonate | 27731 | 0.75 |
| methylmalonate (MMA) | 1496 | 0.6 |
| 1,2-dipalmitoyl-GPC (16:0/16:0) | 19130 | 0.79 |
| 1-palmitoyl-2-stearoyl-GPC (16:0/18:0) | 52616 | 0.79 |
| sphingosine 1-phosphate | 34445 | 0.87 |
| trigonelline (N'-methylnicotinate) | 32401 | 0.26 |
| bilirubin (Z,Z) | 43807 | 1.59 |
| 3-hydroxyindolin-2-one | 42561 | 0.62 |

Supplemental Table 1: List of significantly different metabolites comparing HNG and SHLP2 treated mice.
